# A Snapshot of the Population Structure of *Branchiostoma lanceolatum* in the Racou Beach, France, during Its Spawning Season

**DOI:** 10.1371/journal.pone.0018520

**Published:** 2011-04-15

**Authors:** Yves Desdevises, Vincent Maillet, Michael Fuentes, Hector Escriva

**Affiliations:** CNRS UMR 7232, UPMC Univ Paris 06, Observatoire Océanologique, Banyuls-sur-Mer, France; Centre for Genomic Regulation (CRG), Universitat Pompeu Fabra, Spain

## Abstract

A methodology for inducing spawning in captivity of the lancelet *Branchiostoma lanceolatum* has been developed recently with animals collected at the Racou beach, in the southern coast of France. An increasing amount of laboratories around the world are now working on the evolution of developmental mechanisms (Evo-Devo) using amphioxus collected in this site. Thus, today, the development of new aquaculture techniques for keeping amphioxus in captivity is needed and the study of the natural conditions at which amphioxus is exposed in the Racou beach during their spawning season becomes necessary. We have investigated the amphioxus distribution, size frequency, and population structure in the Racou beach during its natural spawning season using multivariate methods (redundancy analysis and multiple regression). We found a clear preference of amphioxus for sandy sites, something that seems to be a general behaviour of different amphioxus species around the world. We have also estimated the amphioxus growth rate and we show how the animals are preferentially localized in shallow waters during April and June.

## Introduction

Amphioxus (or lancelets) from the subphylum Cephalochordata have long been considered interesting organisms due to their phylogenetic position close to the vertebrates [1], [2]. Recent studies have shown that amphioxus are in fact basal chordates, urochordates being the closest extant relatives to vertebrates [3]. However, in spite of its basal position within the chordate lineage, at the anatomical, genetic, and genomic levels, amphioxus is vertebrate-like but simpler. Both amphioxus and vertebrate embryos have a dorsal hollow nerve cord, segmentally repeated trunk muscles, a notochord, and a pharynx perforated with gill slits. Moreover, the amphioxus genome includes representatives of nearly all the vertebrate gene families, but has fewer gene duplications because it diverged from the vertebrate lineage before the two rounds of complete genome duplication occurred [4], [5]. These anatomical and genomic characteristics have rendered amphioxus an invaluable animal model for studies on the evolution of developmental mechanisms during the invertebrate chordate-to-vertebrate transition [1], [2].

Amphioxus is widespread in tropical and temperate seas and the adult lives burrowed in the sand, gravel, or shell deposits. Traditionally, myomere counts, counts of fin chambers, the position of the atriopore, the position of the anus, and other qualitative differences in notochord and caudal fin shape, have been used to distinguish up to 23 species within the genus *Branchiostoma* [6]. However, to date, developmental studies have been carried out only on three amphioxus species (the European *Branchiostoma lanceolatum*, the East Asian *B. belcheri*, and the Floridian-Caribbean *B. floridae*). To obtain amphioxus embryos, adult animals are collected from the field during their ripe season and allowed (or stimulated) to spawn in the laboratory. In any given year, dates of laboratory spawning have been limited by two factors. First, natural populations of these three most studied species of amphioxus are ripe, at most, for only two to four months each year and, second, even when apparently ripe, animals spawn only at unpredictable intervals of several days. Induction of spawning is not possible for the Asian species (*B. blecheri*) and is possible in the American species (*B. floridae*) only on the natural spawning days (once every 7–10 days) by an electric shock [7]. However, a few years ago, a method for inducing spawning on a daily basis (during the natural spawning season) was developed for the European species *B. lanceolatum* [8], [9]. Since then, several research teams have started to work on the European species and particularly on the amphioxus collected at the Racou beach in southern France.

Even if some studies on the amphioxus ecology have been performed, particularly for *B. nigeriense* on the west coast of Africa [10], [11], and for *B. floridae* from Tampa Bay, Florida [12], [13], very few data exist on the distribution, habitat preference and ecology of *B. lanceolatum*, particularly at the Racou beach location [8], [9], [14], [15]. Reliable ecological information, particularly during the natural spawning season, may be instrumental for the development of adapted culture techniques in the laboratory. This work describes some aspects of the population structure, the spatial distribution, and the relationships between biotic and non-biotic characteristics for *B. lanceolatum* in the Racou beach during this important period of the amphioxus life cycle. We show a clear preference of the animals for sandy sites and we describe how the amphioxus population is structured. We also estimate the lifespan of the amphioxus as well as their growth rate both in captivity and in the wild.

## Materials and Methods

### Study area and amphioxus collection

The study was conducted in the Racou beach close to Argelès-sur-Mer, France, since previous studies showed the presence of an important amphioxus population at this location [9], [15]. Collection was performed with a research boat from the “Laboratoire Arago, Observatoire Océanologique, Université Pierre et Marie Curie/C.N.R.S,” Banyuls-sur-Mer, France. Bottom sediment grab samples containing the amphioxus were collected using a Van Venn-type dredge [9]. The maximum surface and volume covered by the Van Venn dredge was 0.1024 m^2^ and 30 l. Adult amphioxus (>30 mm) were collected from the sand by sieving and small juveniles (<30 mm) by treating the sand in a known volume of sea water with a 10^−4^ dilution of clove oil (85–95% eugenol), an anaesthetic from which the animals recover when returned to sea water [16]. This treatment anesthetizes the animals allowing easy collection from the supernatant water with a 0.2 mm sieve. Once animals (both adults and juveniles) were collected from the sand they were placed in classical culture conditions until further manipulation [8], [9], [17]. Then, amphioxus were counted and total length was measured. The volume of sand was also measured and density of the population was calculated both as individuals/litre of sediment and as individuals/m^2^.

### Sampling design

**Figure 1:**
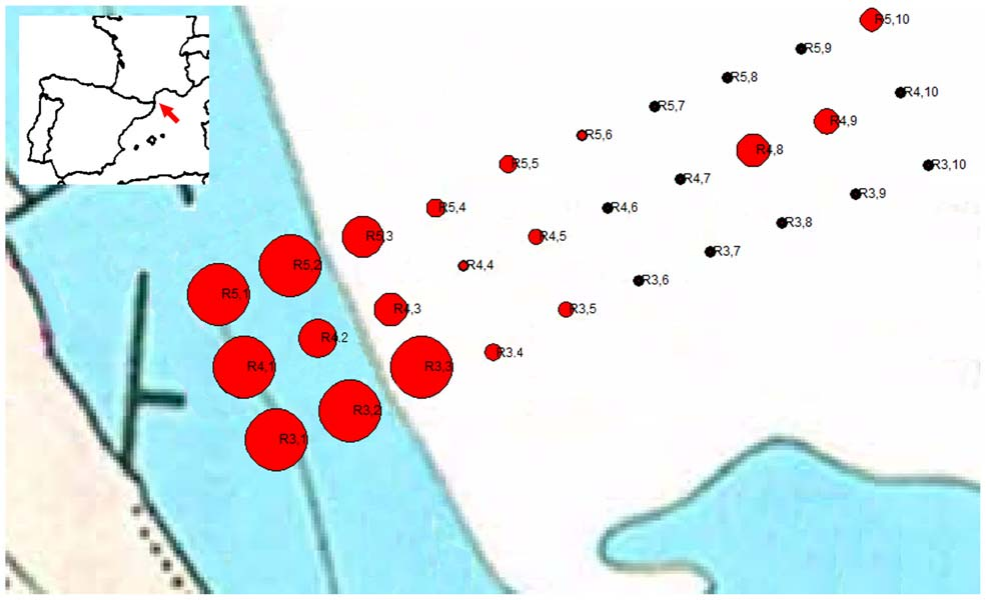
Study area showing the position of the sampling stations. The inset map shows the location of the sampling stations in the Racou beach, France. The size of each red circle is proportional to the density of amphioxus individuals (in ind./l). Stations predominantly composed by silt with a total absence of amphioxus individuals are shown as black dots.

The diversity and distribution of amphioxus was assessed by sampling multiple sites (a total of 50) every 100 m along transects separated by 100 m starting from the site R5.1 (42°32′54″N and 03°03′688″E) in a total surface of 360000 m^2^ (see Fig. 1). Transects were oriented north-east in order to be perpendicular to the sea shore, in an angle of 62° with the azimuth. The number of transects was determined from prior knowledge of the studied area. The southern limit of the studied area was established by the presence of an area of rocks and *Posidonia* sp., and north-east limit was established by the presence of an area of mud (15 extra points were sampled in this area and confirmed the absence of amphioxus in mud, data not shown). Transects have been placed on a map and marked in the field using a global positioning system (GPS). Amphioxus sampling was conducted twice, a first sampling at the end of April 2008 (24^th^ April) and a second sampling at the end of June 2008 (19^th^ June) for radials R3 to R5, but only once for radial R1 and R2 (on 24^th^ April 2008). Since data were collected twice only in radial 3, 4 and 5, we decided to perform our analyses only on these three radials (Fig. 1 shows radials where samples were obtained twice).

### Amphioxus culture in captivity

Amphioxus were induced to spawn in captivity in June 2007 by the temperature shock method [9]. Amphioxus embryos were cultured in Petri dishes until they were 1 month old (8–10 gill slits) at a constant temperature of 20°C and daily feeding with 30,000–80,000 cells/ml/day of mixed algae (1/3 of *Dunaliella tertiolecta*, 1/3 of *Isochrysis galbana*, and 1/3 of *Tetraselmis suecica*). One-month old larvae were then changed to a 50 l aquarium with sand (temperature ranged between 10°C in winter to 21°C in summer) and with the same feeding conditions. 1/3 of the water volume was changed twice a week and larvae present in the changed water were counted, measured and reintroduced into the aquarium. Following metamorphosis (6–7 weeks post-fertilization), the larvae migrated into the sand. Once per month, the larvae were collected from the sand (the small volume of sand in the tank allowed collection of juveniles without the need of an anaesthetic), counted, measured and reintroduced into the aquarium.

### Non-biotic variables

Temperature measurements were performed with a thermo data logger (HOBO Pendant Temp/Light Intensity Data logger, ONSET Computer Corporation, Bourne, MA). This battery-powered thermometer can report 6,500 consecutive data records. The initial settings, including measurement intervals and data acquisition, were set with a computer using the software platform HOBOwares (V. 2.1.1_18), ONSET Computer Corporation. Salinity measurements were determined using a salinity refractometer (American Optical Corp., Buffalo, N.Y.). Particle size analysis was carried out with two different methods: (i) by using a Mastersizer 2000 (Malvern Co. Ltd, UK) whose analysis range is between 0.2 and 2000 µm, and (ii) by sieving dried samples (72 hrs at 65°C) when the sample size was higher than 1600 µm. Depth measurements were performed with a calibrated echosounder (Furuno FCV582L).

### Data analysis

Calculation of grain size parameters was performed as proposed by Folk And Ward [18]. Four parameters were calculated: mean (M = (Φ_16_+Φ_50_+Φ_84_)/3) and median value (Φ_50_ or d(0.5)), standard deviation (σ_1_ = (Φ_84_−Φ_16_)/4+(Φ_95_−Φ_5_)/6.6), skewness (Sk_1_ = (Φ_16_+Φ_84_−2Φ_50_)/2(Φ_84_−Φ_16_)+(Φ_5_+Φ_95_−2Φ_50_)/2(Φ_95_−Φ_5_)) and kurtosis (Kg = (Φ_95_−Φ_5_)/2.44(Φ_75_−Φ_25_)). These calculations allowed us to classify sediments into three major classes, as proposed by Cailleux [19]: gravel (>1250 µm), sand (200–1250 µm) and silt (0–200 µm). Temperature and salinity were not used in our analyses due to their constant values throughout the 50 studied sites (i.e. temperature 15±0.3°C in depth; salinity 36–37 psu). Counts of individual amphioxus at sampling sites were used to build the amphioxus abundance matrix.

To identify environmental variables linked to the abundance and size of amphioxus in the study area, biotic variables (size, density, and number of individuals for juveniles and adults) were related to non-biotic variables (particle size -sand, silt, gravel- and depth) using redundancy analysis (RDA). Redundancy analysis is a direct gradient method (constrained ordination) that can be described as a series of multiple linear regressions of each response variable on explanatory variables, followed by a principal component analysis on fitted values [20]. The significance of the RDA (to assess if environmental variables account for a significant portion of the variation of the biotic variables) was carried out *via* a permutation procedure. The same procedure was used to test the significance of the canonical axes. RDA and the tests of significance were computed using the functions ‘rda’ and ‘anova.cca’ from the ‘vegan’ library [21] of the R statistical language [22]. Amphioxus size (on individuals) and density (on stations) were also separately studied using multiple regressions against environmental variables (depth and particle size (silt, sand, gravel)) followed by a forward selection of significant explanatory variables (see [20]). Due to non normality of variables, permutational testing were performed using the ‘multRegress’ and ‘forward.sel’ functions in R, written respectively by P. Legendre and S. Dray (available at http://www.bio.umontreal.ca/legendre/indexEn.html). Age and growth in length of cohorts of amphioxus was calculated using the successive maximum method [23]. All tests were performed using permutational procedures with 999 permutations.

## Results and Discussion

### Environmental characteristics

The slope of the bottom is the same for the five radials in the study area. The specific depth of each station is shown in Table S1. Tides are not significant in the Racou beach and depths are constant at each point during the day. Thus the closest points to the seashore (R1.1 to R5.1) (see Fig. 1 for R3.1 to R5.1) were at 5 m deep and the five most distant points to the seashore (R3.10 to R5.10) at 18 m deep. However the slope is not constant through the radials. Thus slope is 4% from 150 to 400 m away from the seashore and then decrease to 0.2% from 400 to 600 m away from the seashore.

A zone predominantly composed by silt (Φ_50_ = 100.8±19.8 µm) is present from 15 m to 18 m deep (sites R1.2–1.7; R1.9–1.10; R2.5–2.10; R3.6–3.10; R4.6–4.7; R4.10; R5.7–R5.9) (see black dots in Fig. 1). Since no amphioxus were found in these silty sites, they were excluded from our analyses (see below). Sandy sediments (Φ_50_ = 834.1±65.8 µm) are present in two zones, from the seashore (4 m deep) to 15 m deep and a second zone in the middle of the silt zone at 18 m deep (sites R1.8; R2.1–2.4; R3.1–3.5; R4.1–4.5; R4.8–4.9; R5.1–R5.6) (red dots in Fig. 1). Finally, a small gravel zone (Φ_50_ = 1440.6 µm) is present in only one of the 50 studied points (R5.10) at 18 m deep. These sites have been called sandy and gravel sites because they are predominantly composed by sand and gravel respectively. However, low amounts of silt are always present at each site. The granulometry changed only in a single point between April and June, R3.5, which presented a sand profile in April (Φ_50_ = 891.2 µm) and a silt profile in June (Φ_50_ = 138.4 µm). This change may be explained by sediment dynamics due to short-term processes, such as floods and storms, which are quite usual through the SW Gulf of Lions, including the Racou beach [24], [25].

Small variations of temperature were detected with maximum differences of 1.2°C, ranging from 14.9°C in depth to 16°C at the surface, but no variations were detected in depth between the different sites. Salinity was quite constant throughout the 50 studied points with small changes ranging from 36 to 37 psu.

### Distribution and size frequency of amphioxus

Since aquaculture techniques try to reproduce the conditions present in nature close to the spawning season (which expands from mid-May to mid-July [8], [9]), two quantitative samplings were performed at the Racou beach, with a total sample size of 1106 amphioxus. The first one in April 2008, just before the natural spawning season starts (626 collected animals) and the second in June 2008, within the natural spawning season (480 collected animals), (see Table S1). The geographic and quantitative distribution of the amphioxus within the studied zone is shown on Fig. 1 (for transects R3–5) with a total surface colonized by amphioxus in the Racou studied zone of 20000 m^2^. In April, the highest density of animals was found at station R3.2 with 11.4 ind/l or 869 ind/m^2^, and the lowest was found at R5.10 with 0.7 ind/l (48 ind/m^2^). In June the population structure was similar, with the highest density of animals found at stations R5.1, R4.1 and R3.2 (15.1 ind/l or 731.5/ind/m^2^), and the lowest at R5.10 with 0.85 ind/l (53.3 ind/m^2^). The average density of animals in the studied zone in April and June was 5.4 ind/l or 234.5 ind/m^2^ and 6.5 ind/l or 255.1 ind/m^2^ respectively (density at each station is indicated in Table S1). The amphioxus density in the Racou beach has already been reported twice, by Monniot [15] and by Fuentes et al [9], with 20 ind/l and 1–5 ind/l respectively. These differences may be explained by sampling differences (both in terms of sampling techniques or collection sites), or by differences in the population between different years. Other studies on sites colonized by *B. lanceolatum* have shown important density differences, from 400 animals/m^2^ in Helgoland [26] to 20 animals/m^2^ in the coastal waters of Netherlands [27]. Density of other amphioxus species in different locations is also highly variable, with a highest recorded density of 9000 animals/m^2^ in the west African coast for *B. senegalense* [28]. One interesting point is that our study and the study by Fuentes et al. [9], were performed at the same period of the year, between April and July, which corresponds to the natural spawning season [8], [9]. In contrast, Monniot [15] did not specify the period of the year at which her study was performed. Density changes at the same location depending on the period of the year have been described for *B. caribaeum* [29] and *B. lanceolatum* at Helgoland [26]. Webb suggested that the reason for these changes was probably related to the permeability of the substrate, with amphioxus moving away if the deposit becomes unsatisfactory [26]. Since mobility and other biological activities (i.e. growth, respiration, feeding) of amphioxus change with temperature, Webb suggested that in winter, low temperature inducing low metabolic rate allows amphioxus to live in finer sands, while in summer, higher temperatures increase the amphioxus metabolic rate and induce their migration to coarse or medium grade deposits [26], but this suggestion cannot be applied to *B. floridae*, since it lives in fine sand even in summer with temperatures around 30°C. Thus, an annual study of the *B. lanceolatum* distribution in the Racou beach in order to find possible changes in their distribution would be highly informative.

**Figure 2:**
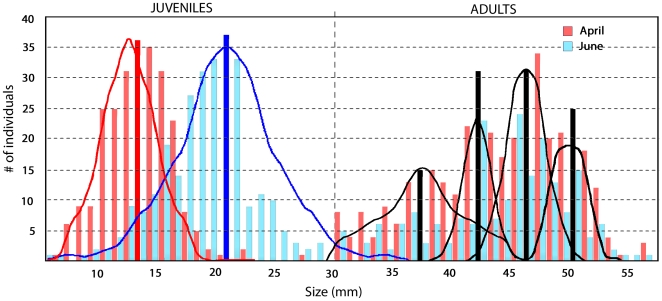
Size frequency distribution computed from data sampled in April (in pink) and June (in light blue). Solid bars indicate the mode of each peak, in red the first peak in April, in blue the first peak in June, in black 2nd–5th peaks in both April and June. Frequency distribution of adult animals (>3 cm long) was similar in April and June.

Body length was measured for all the collected amphioxus. The bimodal frequency distribution (Fig. 2) suggests that the whole population can be divided into two major groups. The first group represents 39.1% and 58.8% of the whole population in April and June respectively and contains juveniles of less than 3 cm long, (our unpublished observations show that ripe animals are always longer than 3 cm [8], [9]). The second group contains adults (longer than 3 cm, Fig. 2) (the number of adults/juveniles at each station is shown in Table S1). Several studies have estimated the lifespan of different amphioxus species. Wells [30], Nelson [31], and Futch & Dwinell [32] suggested a 2 to 3 yrs lifespan for *B. floridae* based on the apparent number of generations present in the sampled size-frequency histograms. Chin [33] and Chen [34] suggested a maximum age of 2 to 3 yrs for *B. belcheri*. Gosselck & Spittler [35] estimated 4–5 years life-span for *B. senegalense* off North-West Africa and a considerably longer lifespan of 8 yrs was suggested by Courtney [36] for *B. lanceolatum* living in the relatively cold waters off Helgoland. For *B. lanceolatum* in the Racou beach, we have constructed length-frequency histograms showing the presence of 5 modal lengths of 12, 36, 41, 48 and 50 mm in April (Fig. 2 blue bars). A similar distribution into five modal lengths was also found in June (Fig. 2 red bars). Since the spawning season spans from May to July and considering that it has been proposed that amphioxus grow continuously during their entire life [13], these sizes may represent the average size of animals of 1 to 5 years old. Thus, *B. lanceolatum* lifespan at the Racou can be estimated to 5 years.

**Figure 3:**
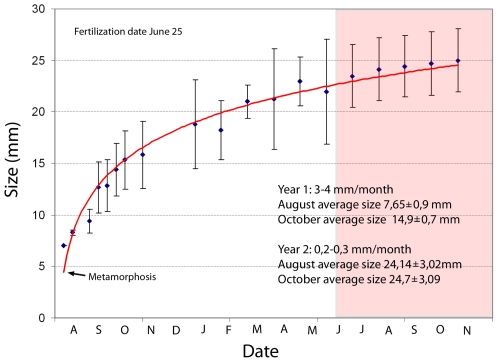
Growth curve of amphioxus larvae in captivity at room temperature (from 10°C in winter to 21°C in summer). The white part of the graph corresponds to the first year (animals were fertilized in late June 2007 and the graph starts when these embryos become post-metamorphic larvae in August 2007) and the pink side to the second year (2008). A minimum of 10 larvae were measured for each point (error bars = standard deviation from the mean).

An interesting point is that the juvenile population (i.e. less than 3 cm long) increased their average size between April and June from 11.9±1.1 mm to 18.4±1.9 mm (compare the first blue and red peaks in Fig. 2), indicating that the growth rate of juveniles during April/June 2008 in the wild was 3–4 mm/month. The amphioxus growth rate in captivity (Fig. 3) also shows a growth rate of 3–4 mm/month for amphioxus larvae with a similar size. Previous studies on *B. floridae* showed that growth rate for juveniles was 6 mm in 12 days from the 18^th^ to the 30^th^ of July [13], indicating a clear difference between these two amphioxus species. *In vitro* comparisons of embryonic development at different temperatures also showed a higher growth rate for *B. floridae* than for *B. lanceolatum* [8]. Moreover, in a study of the age structure of *B. lanceolatum* in the North Sea off Helgoland (0 to 17°C yearly temperature range) and in the warmer water at the Racou (10 to 24°C yearly temperature range), Courtney reported initial growth rates of 10 and 20 mm/yr, respectively, with faster growth during the warmest months of the year [36]. This would mean that the first cohort in Fig. 2 corresponds, as we have assumed, to the 1 year old juveniles (less than 20 mm long). The lack of growth between April and June in the second group of amphioxus composed of adults (longer than 3 cm, data not shown) may reflect a logarithmic decrease of growth with age, something that has been suggested for *B. lanceolatum* and other amphioxus species [35], [36]. In this sense, amphioxus grown in captivity support this assumption since they show a clear decrease in the growth rate with time (from 3–4 mm/month in postmetamorphic animals to 0.2–0.3 mm/month in one year old juveniles) (see Fig. 3). Other possibilities may also explain differences in growth rate. Thus, Webb observed differences in the growth rate between nearby populations of *Branchiostoma nigeriense* [37] in Lagos, Nigeria. A population living within the lagoon grew 30 mm in three months (January–March) while two other populations closely located, one within the connexion between the lagoon and the open sea, and the second in the open sea, grew only 7 and 5 mm respectively in the same period. Webb interpreted these differences by the different food availability in these three different locations. Future laboratory experiments testing amphioxus growth at different temperatures and with different food regimes should shed light on the specific requirements for different growth rates of the same amphioxus population.

### Habitat preference

**Figure 4:**
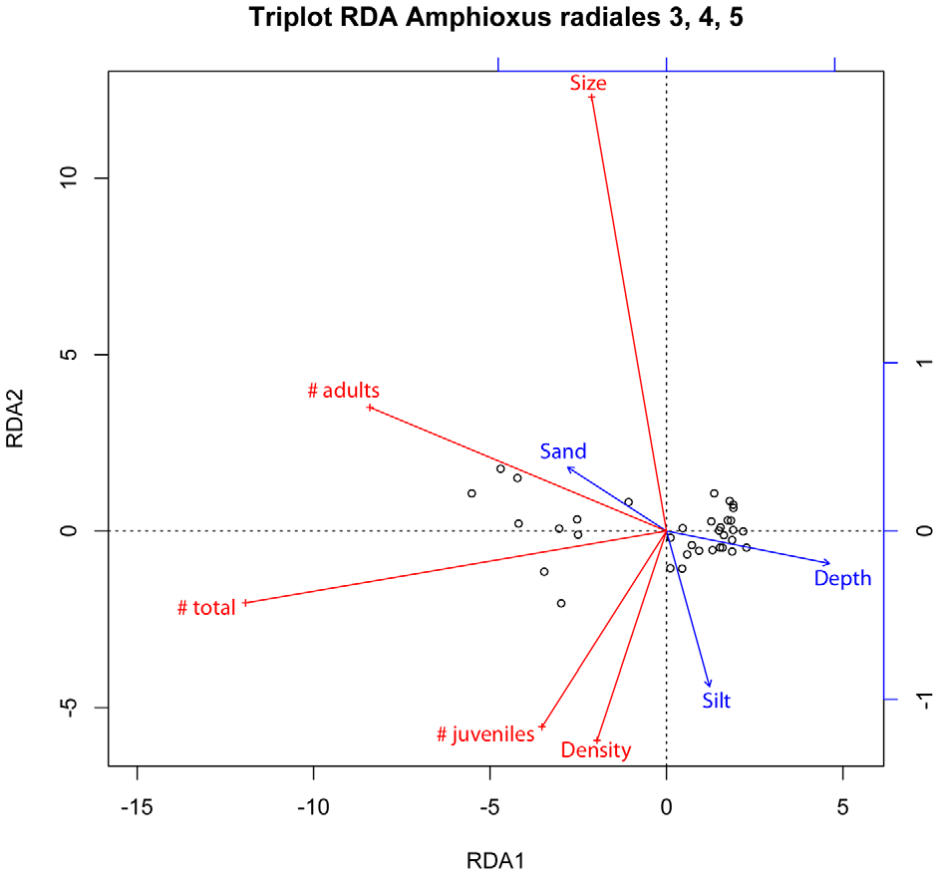
Triplot showing the result of the redundancy analysis of amphioxus size, number (of adults, juveniles, and all individuals) and density (in ind./l) constrained by environmental variables (percentage of sand and silt, and depth). Canonical axes 1 and 2 are represented (RDA1 and RDA2), accounting in total for 63.5% of the variation of the original dataset.

Redundancy analysis (RDA) assesses how biotic factors of the amphioxus population (mean size, density, and abundances of juveniles (<30 mm), adults (>30 mm), and all individuals) are explained by environmental variables (depth and sediment granulometry). Even if temperature as well as salinity changes have been shown to be very important in the life cycle of other amphioxus species [11], [38], [39], we have not included these two environmental variables in the analysis because both were constant during this study. In fact, this study represents a snapshot of a precise moment in the year and it is not a dynamic view of the amphioxus population during its life cycle. Results are shown using a triplot representation (Fig. 4), displaying stations (Fig. 4, in black), response biological variables (Fig. 4, in red), and explanatory environmental variables (Fig. 4, in blue). We only used in these analyses stations were amphioxus were detected. Stations predominantly composed by silt (sites R1.2–1.7; R1.9–1.10; R2.5–2.10; R3.6–3.10; R4.6–4.7; R4.10; R5.7–R5.9), with a total absence of amphioxus, were excluded (see above). Temperature and salinity were excluded from these analyses due to their constant values at all sites. The gravel vector was also excluded from the analysis because it was almost perfectly negatively collinear to the sand vector. The RDA was globally significant (constrained (e.g. canonical) variation is 63.5%, P = 0.001) as well as the first canonical axis (accounting for 99.99% of the canonical variation, P = 0.001).

The triplot representation shows that the number of adults is strongly negatively correlated to depth (which is the main variable structuring the dataset, close to canonical axis 1), as well as the number of juveniles but in a weaker manner. The number of adults and total number of individuals are positively correlated to sand. However, while the number of adults is weakly negatively correlated to silt, juvenile abundance and density are positively correlated to silt (i.e. here it should be noted that the silt variable indicates the presence of low amounts of silt in a sample composed predominantly by sand). This is coherent with the positive link (P = 0.01) of size with sand (adult animals are longer and prefer sand) while size is negatively correlated to silt and density (juvenile animals are smaller and in higher density). The variable selection procedure used in multiple regressions always selected depth among the significant explanatory variables accounting for variations in body size and density, with which depth was always negatively correlated (Table 1). Silt was also selected as positively linked to size for juveniles (P = 0.01) and all individuals (P = 0.02), as well as density (P = 0.03). These results suggest a clear preference of amphioxus, particularly adult animals, for sandy sites in the Racou beach. Taking into account the total absence of amphioxus (both adults and juveniles) in silt, the positive link between both the number of juvenile animals and density with silt does not necessarily indicate a juvenile preference for silty sites (i.e. silt is always present in low amounts). However, the differences observed between adults and juveniles may indicate a higher tolerance for the presence of silt by juveniles than by adults. The importance of sediment type in the distribution of amphioxus has been observed repeatedly. Webb and Hill [10] showed that *B. nigeriense* preferred sand with less than 25% fine grains and a low silt content, Boschung and Gunter [40] found *B. caribaeum* in Mississippi Sound on coarse sand 90% of the time, rarely on fine sand, and never on clay, Cory and Pierce [41] also found *B. caribaeum* from South Carolina to Georgia in coarser sand and no specimens were found in silt, and Gosselck [35] found *B. senegalense* in the off-shore shelf region off North West Africa in sediments similar to gravel and mixtures of coarse sand and gravel but never in mud. Webb and Hill [10] conducted an interesting experiment by placing amphioxus (*B. nigeriense*) of different sizes in a number of graded sands (from 2 to 0.1 mm). They showed that animals placed in coarse and medium-size sands survived either burrowed in the sand or lying on the surface, but those placed in fine sand and silt died with an accumulation of sand particles in the gut and with the oral aperture blocked by a mass of grains. They concluded that amphioxus avoid fine sand bottoms due to occlusion of the oral aperture and the atriopore. Moreover, the permeability of the bottom type and the presence of circulating water between the sand grains are also controlling factors for the preference of amphioxus for sand and coarse sand. Our results also show a clear preference of *B. lanceolatum* in the Racou beach for sandy sites, suggesting that most if not all the different amphioxus species in the world need such a permeable bottom and avoid fine grains that may block their oral aperture. *Branchiostoma floridae* seems to be an exception to this rule since they live in fine sand bottoms that evidently do not block their oral aperture.

**Table 1:** Result of multiple regression of a response variable (body size or density) against explanatory environmental variables (depth, silt, sand, and gravel).

Response variable	Age	Sampling period	Significant explanatory variables (partial regression coefficient and p-value)	Slope of relationship with depth
		All	Depth (−1.77, 0.001), Silt (9.74, 0.02)	−1.60
	All	April	Depth (−1.90, 0.001), Silt (26.10, 0.002), Sand (36.41, 0.001)	−2.47
		June	Depth (−1.44, 0.001), Sand (−16.65, 0.008)	−0.88
		All	Depth (−0.46, 0.001), Silt (4.61, 0.01)	−0.35
Body size	Juveniles	April	Sand (3.99, 0.03)	0
		June	Depth (−0.22, 0.001)	−0.22
		All	Depth (−0.58, 0.001)	−0.58
	Adults	April	Depth (−0.61, 0.001)	−0.61
		June	Depth (−0.59, 0.001)	−0.59
		All	Depth (−0.93, 0.001), Silt (9.75, 0.03)	−0.76
Density	All	April	Depth (−0.55, 0.001)	−0.55
		June	Depth (−0.96, 0.003)	−0.96

Results were computed for the complete dataset, and for different sub-datasets, considering different combinations of age (adult and juvenile) and sampling period (April and June). Significant explanatory variables were selected using a forward selection procedure and tested by permutations.

**Figure 5:**
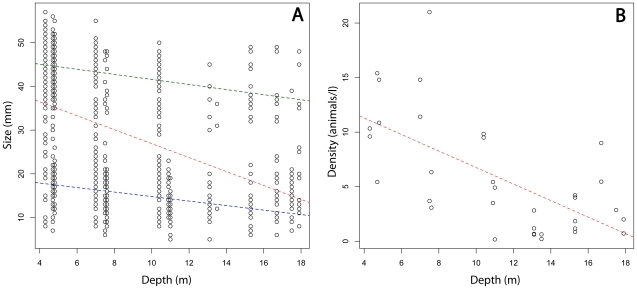
Bivariate linear relationships between amphioxus and depth. A: Linear relationships between amphioxus individual size and depth for juveniles (green line), adults (blue line) and all individuals (red line). B: Linear relationships between individual density and depth for all stations.

As previously reported by Webb at the Racou [14], our RDA analysis shows a strong negative correlation between the presence of adults and depth but only a weak negative correlation between the presence of juveniles and depth (Fig. 4). However, bivariate linear relationships between amphioxus individual size and depth show no differences (compare green and blue lines in Fig. 5A and Table 1). This difference may be explained by the fact that bivariate linear analyses do not consider the multiplicity of interactions between all variables like RDA do. In any case, these results suggest that amphioxus are always concentrated in shallow waters close to the seashore. This is very clear on Fig. 5B showing the linear relationship between density and depth. Reasons for this distribution are not clear, thus Webb interpreted this fact by the need for a copious stream of interstitial water in adults (they pass a greater volume of water through the pharynx) but not in juveniles. Webb suggested that as depth increases, the deposit becomes finer and less permeable hampering the presence of adults [14]. Gosselck also observed higher densities of amphioxus (*B. senegalense*) in shallow waters of North West Africa [28] (never deeper than 40 m), and he attributed this distribution to the presence of detritus in deep waters that may block the water stream required by the filter feeding animals. But this distribution could also be related to the spawning season since seasonal changes in the amphioxus distribution in a given site have been described [26], [29] and only sampling throughout the year will help to answer this question.

### Conclusions

The present study provides some new knowledge on amphioxus distribution during their spawning season within the Racou beach in southern France, a place where amphioxus are usually collected for Evo-Devo studies by different laboratories around the world. Amphioxus is now an animal model for many researchers and the publication of recent studies concerning its life cycle, reproduction in captivity, and aquaculture, shows the growing interest of the scientific community for the biology of this animal [7], [8], [9], [17], [34], [42], [43], [44], [45], [46]. In the present preliminary work, we have established that it exists a clear preference of amphioxus for sandy sites and their complete absence in silt. This result is in accordance with many other studies on other amphioxus species [10], [21], [28], [37], suggesting that preference for coarse sediments is a general behaviour of amphioxus species, even if some exceptions exist since *B. floridae* lives in fine sand habitats. Several studies have also shown a negative correlation between amphioxus presence and the organic matter content [11], [47]. In this study we did not controlled the presence of organic matter in the sand, and future studies will be necessary to confirm this correlation as a general fact for amphioxus species. We also describe a clear structure of the adult population at the Racou beach. Indeed, amphioxus are preferentially localized in shallow waters close to the seashore. This preliminary study represents an ecological snapshot of the amphioxus population in the Racou beach because the collection of animals was performed uniquely during April and June 2008, a period extremely important in the amphioxus life cycle since it lies at the beginning of the natural spawning season [9]. Since observations performed by others [26], [29] showing population fluctuations during the year suggest that the population structure observed in this study may change depending on the moment of the year, further studies throughout the whole annual cycle over several successive years would therefore be highly informative to establish the dynamics of the population structure during the year. Finally, our study, even if concentrated on two months, represents a first approach towards a comprehension of possible ecological factors affecting the reproductive behaviour of amphioxus, since adults close to the spawning season (possessing ripe gonads) prefer lower depths and specific sand granulometry. Further studies on the physical and biological components of the sites where adult amphioxus preferentially live may help to further develop aquaculture facilities allowing development and maintenance of whole life cycle of *B. lanceolatum* in captivity.

## Supporting Information

Table S1 Data obtained from the different sites where amphioxus have been found. Sites containing only silt, where amphioxus were absent, have not been included. The total number of animals per site, both juveniles and adults, is indicated as well as their density (both as number of animals/m2 and number of animals/l) and average size. Numbers of adults (>3 cm long) and juveniles (<3 cm long) are also included. The percentage of silt, sand and gravel in each site is indicated as well as the depth and the collection date. ND: not determined.
(DOC)
