# Supplementary material for: A Snapshot of the Population Structure of Branchiostoma lanceolatum in the Racou Beach, France, during Its Spawning Season
Source: PLoS One. 2011 Apr 15;6(4):e18520. doi: 10.1371/journal.pone.0018520 (PMC3078106; doi:10.1371/journal.pone.0018520)
Supplement: Table S1 — Data obtained from the different sites where amphioxus have been found. Sites containing only silt, where amphioxus were absent, have not been included. The total number of animals per site, both juveniles and adults, is indicated as well as their density (both as number of animals/m2 and number of animals/l) and average size. Numbers of adults (>3 cm long) and juveniles (<3 cm long) are also included. The percentage of silt, sand and gravel in each site is indicated as well as the depth and the collection date. ND: not determined. (DOC) [file pone.0018520.s001.doc]

| Site | Density  (nb/m²) | Density  (nb/L) | Total  number | Average  Size | Adults  number | Juveniles  number | Silt | Sand | Gravel | Depth | Date  (2008) |
| --- | --- | --- | --- | --- | --- | --- | --- | --- | --- | --- | --- |
| R1.1 | 380.85937 | 6.96428571428571 | 39 | 39.974358974359 | 35 | 4 | ND | ND | ND | 5.2 | 25/04 |
| R1.8 | 185.54687 | 3.58490566037736 | 19 | 15 | 3 | 16 | ND | ND | ND | 17.4 | 25/04 |
| R2.1 | 126.95312 | 13 | 13 | 38.9230769230769 | 10 | 3 | 0.002986952 | 0.974689514 | 0.02232353 | 4 | 25/04 |
| R2.2 | 341.79687 | 10.6060606060606 | 35 | 27.3428571428571 | 19 | 16 | 0.00071398 | 0.988147937 | 0.01113808 | 8.2 | 25/04 |
| R2.3 | 126.95312 | 8.66666666666667 | 13 | 19.6923076923077 | 3 | 10 | 0.003189571 | 0.931354874 | 0.06545555 | 11.4 | 25/04 |
| R2.4 | 175.78125 | 3 | 18 | 17.8333333333333 | 2 | 16 | 0.008511252 | 0.85660704 | 0.13488171 | 14.4 | 25/04 |
| R3.1 | 703.125 | 9.6 | 72 | 42.2361111111111 | 66 | 6 | 0.002986952 | 0.974689514 | 0.02232353 | 4.3 | 25/04 |
| R3.1 | 605.46875 | 10.3333333333333 | 62 | 21 | 50 | 12 | 0.002986952 | 0.974689514 | 0.02232353 | 4.3 | 20/06 |
| R3.2 | 869.14062 | 11.4102564102564 | 89 | 38.685393258427 | 75 | 14 | 0.00071398 | 0.988147937 | 0.01113808 | 7 | 25/04 |
| R3.2 | 722.65625 | 14.8 | 74 | 21.8783783783784 | 7 | 67 | 0.00071398 | 0.988147937 | 0.01113808 | 7 | 20/06 |
| R3.3 | 556.64062 | 9.5 | 57 | 32 | 37 | 20 | 0.003189571 | 0.931354874 | 0.06545555 | 10.4 | 25/04 |
| R3.3 | 576.17187 | 9.83333333333333 | 59 | 30.3559322033898 | 30 | 29 | 0.003189571 | 0.931354874 | 0.06545555 | 10.4 | 20/06 |
| R3.4 | 58.59375 | 1.17647058823529 | 6 | 21.8333333333333 | 2 | 4 | 0.008511252 | 0.85660704 | 0.13488171 | 13.1 | 25/04 |
| R3.4 | 39.0625 | 0.615384615384615 | 4 | 30.75 | 2 | 2 | 0.008511252 | 0.85660704 | 0.13488171 | 13.1 | 20/06 |
| R3.5 | 87.890625 | 1.83673469387755 | 9 | 19.4444444444444 | 3 | 6 | 0.003540055 | 0.807657008 | 0.18880294 | 15.3 | 25/04 |
| R4.1 | 742.1875 | 10.8571428571429 | 76 | 43.7236842105263 | 75 | 1 | 0.001633755 | 0.953402472 | 0.04496377 | 4.8 | 25/04 |
| R4.1 | 722.65625 | 14.8 | 74 | 22 | 30 | 44 | 0.001633755 | 0.953402472 | 0.04496377 | 4.8 | 20/06 |
| R4.2 | 205.07812 | 3.68421052631579 | 21 | 17.4285714285714 | 4 | 17 | 0.002011937 | 0.970022131 | 0.02796593 | 7.5 | 25/04 |
| R4.2 | 205.07812 | 21 | 21 | 23 | 1 | 20 | 0.002011937 | 0.970022131 | 0.02796593 | 7.5 | 20/06 |
| R4.3 | 136.71875 | 3.5 | 14 | 13.2857142857143 | 0 | 14 | 0.001934719 | 0.973538039 | 0.02452724 | 10.9 | 25/04 |
| R4.3 | 185.54687 | 5.42857142857143 | 19 | 17 | 0 | 19 | 0.001934719 | 0.973538039 | 0.02452724 | 10.9 | 20/06 |
| R4.4 | 29.296875 | 0.625 | 3 | 26.3333333333333 | 2 | 1 | 0.002644582 | 0.881202589 | 0.11615283 | 13.5 | 25/04 |
| R4.4 | 9.765625 | 0.2 | 1 | 13 | 1 | 0 | 0.002644582 | 0.881202589 | 0.11615283 | 13.5 | 20/06 |
| R4.5 | 78.125 | 4.21052631578947 | 8 | 16.5 | 2 | 6 | 0.002552048 | 0.733445265 | 0.26400269 | 15.3 | 25/04 |
| R4.5 | 78.125 | 4 | 8 | 35.25 | 7 | 1 | 0.002552048 | 0.733445265 | 0.26400269 | 15.3 | 20/06 |
| R4.8 | 58.59375 | 5.45454545454545 | 6 | 8.5 | 0 | 6 | 0.54110232 | 0.44643952 | 0.01245816 | 16.7 | 25/04 |
| R4.8 | 263.67187 | 9 | 27 | 24 | 9 | 18 | 0.54110232 | 0.44643952 | 0.01245816 | 16.7 | 20/06 |
| R4.9 | 97.65625 | 2.85714285714286 | 10 | 11.8 | 0 | 10 | 0.54110232 | 0.44643952 | 0.01245816 | 17.5 | 25/04 |
| R4.9 | 97.65625 | 2.85714285714286 | 10 | 20.3 | 1 | 9 | 0.54110232 | 0.44643952 | 0.01245816 | 17.5 | 20/06 |
| R5.1 | 371.09375 | 5.42857142857143 | 38 | 34.2631578947368 | 28 | 10 | 0.001244727 | 0.992185879 | 0.00656939 | 4.7 | 25/04 |
| R5.1 | 751.95312 | 15.4 | 77 | 36.0779220779221 | 49 | 28 | 0.001244727 | 0.992185879 | 0.00656939 | 4.7 | 20/06 |
| R5.2 | 302.73437 | 6.3265306122449 | 31 | 13.9032258064516 | 2 | 29 | 0.01343253 | 0.7591718 | 0.22739567 | 7.6 | 25/04 |
| R5.2 | 224.60937 | 3.06666666666667 | 23 | 25.304347826087 | 7 | 16 | 0.01343253 | 0.7591718 | 0.22739567 | 7.6 | 20/06 |
| R5.3 | 263.67187 | 4.90909090909091 | 27 | 12.037037037037 | 0 | 27 | 0 | 0.8136116 | 0.1863884 | 11 | 25/04 |
| R5.3 | 9.765625 | 0.166666666666667 | 1 | 5 | 0 | 1 | 0 | 0.8136116 | 0.1863884 | 11 | 20/06 |
| R5.4 | 107.42187 | 2.82051282051282 | 11 | 12.4545454545455 | 0 | 11 | 0.004216563 | 0.922162253 | 0.07362118 | 13.1 | 25/04 |
| R5.4 | 48.828125 | 0.666666666666667 | 5 | 17.2 | 0 | 5 | 0.004216563 | 0.922162253 | 0.07362118 | 13.1 | 20/06 |
| R5.5 | 58.59375 | 1.15384615384615 | 6 | 12.6666666666667 | 0 | 6 | 0.007890083 | 0.863147871 | 0.12896205 | 15.3 | 25/04 |
| R5.5 | 29.296875 | 0.857142857142857 | 3 | 29 | 1 | 2 | 0.007890083 | 0.863147871 | 0.12896205 | 15.3 | 20/06 |
| R5.10 | 48.828125 | 0.704225352112676 | 5 | 21.6 | 1 | 4 | 0.007110996 | 0.407007564 | 0.58588144 | 17.9 | 25/04 |
| R5.10 | 117.1875 | 2 | 12 | 23.8333333333333 | 4 | 8 | 0.007110996 | 0.407007564 | 0.58588144 | 17.9 | 20/06 |
